# Parent–adolescent discrepancies in perceptions of parental warmth: Cross‐cultural differences and longitudinal associations with internalizing symptoms

**DOI:** 10.1111/jora.70093

**Published:** 2025-10-31

**Authors:** Concetta Esposito, Maria Concetta Miranda, W. Andrew Rothenberg, Ann T. Skinner, Jennifer E. Lansford, Sevtap Gurdal, Daranee Junla, Paul Oburu, Concetta Pastorelli, Emma Sorbring, Laurence Steinberg, Liliana Maria Uribe Tirado, Saengduean Yotanyamaneewong, Liane P. Alampay, Suha M. Al‐Hassan, Marc H. Bornstein, Lei Chang, Kirby Deater‐Deckard, Laura Di Giunta, Kenneth A. Dodge, Dario Bacchini

**Affiliations:** ^1^ Department of Humanities University of Naples “Federico II” Naples Italy; ^2^ Mental Health Department ASL Napoli 2 Nord Naples Italy; ^3^ Center for Child & Family Policy Duke University Durham North Carolina USA; ^4^ Division of Educational Science and Languages University West Trollhättan Sweden; ^5^ Department of Psychology Chiang Mai University Chiang Mai Thailand; ^6^ School of Education Maseno University Maseno Kenya; ^7^ Department of Psychology Università di Roma “La Sapienza” Rome Italy; ^8^ Division of Social Work and Social Pedagogy University West Trollhättan Sweden; ^9^ Department of Psychology and Neuroscience Temple University Philadelphia Pennsylvania USA; ^10^ Center for Social and Humanities Research King Abdulaziz University Jeddah Saudi Arabia; ^11^ Department of Psychology Universidad de San Buenaventura Medellín Colombia; ^12^ Department of Psychology Ateneo de Manila University Quezon City Philippines; ^13^ Abu Dhabi Early Childhood Authority Abu Dhabi United Arab Emirates; ^14^ Eunice Kennedy Shriver National Institute of Child Health and Human Development Bethesda Maryland USA; ^15^ Institute for Fiscal Studies London UK; ^16^ UNICEF New York City New York USA; ^17^ Department of Psychology University of Macau Macau China; ^18^ Department of Psychological and Brain Sciences University of Massachusetts Amherst Amherst Massachusetts USA

**Keywords:** cross‐cultural, internalizing symptoms, parent–adolescent discrepancy, parental warmth

## Abstract

Research suggests that adolescents often perceive parental behaviors—such as expressions of warmth and affection—differently than their parents do. These parent–adolescent discrepancies offer meaningful insight into family functioning during adolescence and adolescent mental health, though existing findings remain mixed. Grounded in interpersonal acceptance–rejection theory (IPARTheory), this study investigates longitudinal, bidirectional associations between parent–adolescent discrepancies in perceived parental warmth and adolescent internalizing symptoms. The sample included 1219 parent–adolescent dyads (both mothers and fathers) from 12 cultural groups across 9countries, followed across three time points spanning 5 years, with children's mean age being 10.72 years (SD = 0.67) at Wave 1, 13.19 years (SD = 0.90) at Wave 2, and 15.60 years (SD = 0.94) at Wave 3. The results of latent congruence models showed that mothers reported higher warmth than adolescents, whereas no significant discrepancies emerged between fathers and adolescents. The cross‐sectional analyses indicated that a higher parent–adolescent discrepancy in parental warmth perceptions was linked to increased internalizing symptoms in adolescents and lower overall warmth perceived by parents and adolescents in the dyad. However, over the long term, marginal effects were observed only between greater internalizing symptoms in adolescents and lower overall warmth experienced, and vice versa. Additionally, some cross‐cultural differences in the discrepancies between parents and adolescents were identified. These findings highlight the importance of congruence between parents' and adolescents' perceptions of parental warmth, which may play a critical role in reducing adolescent internalizing symptoms, at least in the short term. Future research should deepen these dynamics across different cultures and developmental stages to improve intervention strategies and strengthen family‐based mental health support.

## INTRODUCTION

The quality of the parent–adolescent relationship is a key predictor of adolescent developmental outcomes (Steinberg, 2001). Research has consistently documented strong associations between negative parenting dimensions and psychological problems in adolescents, including internalizing symptoms such as anxiety and depression (e.g., Manuele et al., 2023; Pinquart, 2017).

Interpersonal acceptance–rejection theory (IPARTheory; Rohner, 2004, 2021) provides a valuable framework for understanding the relation between parenting and adolescent development, mainly through its emphasis on parental warmth—defined as expressions of love, affection, and acceptance. According to the theory, parental warmth is beneficial across cultures, as it addresses humans' fundamental need for acceptance from attachment figures (Rohner & Lansford, 2017). Furthermore, the theory emphasizes that children's subjective perceptions of their parents' behaviors, rather than the behaviors themselves, are the most significant predictors of children's adjustment (Rohner, 2021).

A theoretical emphasis on subjective perceptions is supported by extensive empirical evidence showing that adolescents and parents generally report different perceptions of family functioning, including parenting (Jager et al., 2012, 2014; Korelitz & Garber, 2016; Smetana et al., 2006). Specifically, it is estimated that parent–adolescent agreement typically falls in the low‐to‐moderate range (De Los Reyes & Ohannessian, 2016; Hou et al., 2020). Differences in perspectives between adolescents and their parents—commonly referred to as parent–adolescent discrepancies—are not mere measurement errors (De Los Reyes et al., 2022); instead, they provide meaningful insights into parent–adolescent relationships (De Los Reyes et al., 2019; De Los Reyes & Ohannessian, 2016), particularly in the context of family adjustment processes (Mastrotheodoros et al., 2019).

Parent–adolescent discrepancies are acknowledged to contribute to adolescent internalizing symptoms (Human et al., 2016; Ohannessian & De Los Reyes, 2014). Focusing exclusively on mother–adolescent dyads, Nelemans et al. (2023) revealed an inverse relation between such discrepancies and adolescent internalizing symptoms, with internalizing symptoms predicting greater discrepancies over time. This finding supports the depression‐distortion hypothesis (De Los Reyes & Kazdin, 2005), which proposes that internalizing symptoms might influence how family members perceive their relationships, creating a potential reciprocal dynamic between perceptions and psychological adjustment.

Despite the theoretical importance of warmth in parent–adolescent relationships, studies examining discrepancies in perceptions of parental warmth specifically are relatively limited and have provided mixed results. Most research has focused on broader aspects of family functioning, such as parent–adolescent communication or conflict, leaving relatively less attention to how differences in perceptions of parental warmth might impact adolescent adjustment. Although parental warmth is associated with fewer adolescent internalizing problems across different cultural contexts (Rothenberg et al., 2020, 2022), research on the correlates of parent–adolescent discrepancies has predominantly focused on monocultural, Western populations.

The present study addresses these gaps by examining longitudinal and bidirectional relations between parent–adolescent discrepancies in perceived warmth and adolescent internalizing symptoms across 12 cultural groups from 9 countries, involving both mothers and fathers. Specifically, it focuses on parents' and adolescents' perceptions of parental warmth as both a potential outcome of and precursor to adolescents' internalizing problems during early to middle adolescence. Adolescence is particularly relevant as it encompasses normative developmental processes and multiple transitional experiences, such as the onset of puberty and entry to secondary school. Also, by focusing on discrepancies in parental warmth—a theoretically universal construct in IPARTheory—this study also sheds light on how cultural context is associated with the magnitude of such discrepancies. This dual focus enhances our understanding of the association between parent–adolescent discrepancies and adolescent psychological adjustment, providing new insights into the dynamic and culturally specific nature of these relations.

### Parent–adolescent discrepancies in family functioning

Parenting occurs in a shared parent–adolescent context, so one might reasonably expect notable consistency between parents' and adolescents' reports. However, as symbolic interaction theory (Stryker, 1972) suggests, individuals' roles and positions in social structures shape their perceptions of interactions. In the family context, parents and adolescents occupy distinctly different positions with different responsibilities, power dynamics, and developmental tasks. These different roles mean that parents and adolescents may interpret the same interactions in distinct ways, as their positions within the family create varying frameworks for understanding social exchanges. Rather than representing measurement error or one “correct” versus “incorrect” perspective, these discrepancies reflect the authentic lived experiences of family members operating from different structural positions.

Building on this foundation, the modified operations triad model (De Los Reyes & Ohannessian, 2016) provides a more specific framework for understanding informant discrepancies in family research. Adapted from the original Operations Triad Model (De Los Reyes et al., 2013), this model posits that discrepancies between family members' reports represent meaningful information about family functioning. The model identifies three key sources of informant discrepancies: (1) attributional processes—different family members may attribute the same behavior to different causes based on their perspective and motivations; (2) observational perspectives—family members have access to different behavioral information based on their position in the family context; and (3) response biases— systematic tendencies to report in ways that are consistent with one's role or socially desirable from one's position (De Los Reyes & Kazdin, 2005). Importantly, the model argues that these discrepancies can serve as indicators of family relationship quality, with larger discrepancies potentially indicating communication problems, conflict, or lack of shared understanding within the family system.

Together, these theoretical frameworks suggest that examining parent–adolescent discrepancies in perceptions of parenting practices serves multiple important functions: (1) it acknowledges that social experiences are subjective and that different perspectives can all be valid at the same time; (2) it provides insight into family communication patterns and shared understanding; and (3) it may reveal aspects of family functioning that would be missed by relying on single informants. Therefore, studies that specifically examine informant discrepancies in families are crucial for a comprehensive understanding of family dynamics and their associations with adolescent development.

Parent–adolescent discrepancies have been investigated across multiple dimensions, including parent–adolescent communication and family cohesion (e.g., Córdova et al., 2016), conflict (e.g., Mastrotheodoros et al., 2019), parental support (e.g., Mastrotheodoros et al., 2019), positive parenting (e.g., Córdova et al., 2016; Nichols & Tanner‐Smith, 2022), parental control (e.g., Keeley et al., 2024; Xu & Zheng, 2022; Zhai et al., 2025), and parental warmth (Dotterer & Day, 2019; Gniewosz et al., 2023; Hou et al., 2018; Nelemans et al., 2023; Sloan et al., 2025; Wen et al., 2023). Overall, considerable consistency in discrepancies in parent–adolescent reports across various family constructs has emerged (Rote & Smetana, 2016).

That said, two dynamics moderate parent–adolescent discrepancies. One is the developmental trajectory of adolescence, and the other is variation in the direction of report discrepancies, that is, whether the parent or the adolescent report is more positive about family functioning. Although less common, some studies, particularly those using person‐centered approaches, have identified subgroups of families in which a small proportion of children report more positive perceptions of family functioning compared with their parents (Fan et al., 2023; Rote & Smetana, 2016; Wen et al., 2023). For example, Fan et al. (2023) identified that in 7.7% of Chinese parent–adolescent dyads, adolescents reported more positive perceptions of parental emotion socialization than their parents did. Similarly, in a study conducted with Mexican‐origin adolescents, Wen et al. (2023) found evidence of a “Teen High” group, in which adolescents reported receiving higher levels of positive parenting from their mothers than the mothers themselves reported. In both studies, participants were early adolescents, specifically around the age of 11–13 years, and Wen et al. (2023) observed that this pattern did not continue into later adolescent years.

In contrast, most research has generally shown that, on average, parents tend to report slightly more positive parenting practices than their children do (Ohannessian & De Los Reyes, 2014). These discrepancies have been examined through both adaptive and maladaptive models (Ohannessian et al., 2000). The adaptive model emphasizes the developmental trajectory as adolescents navigate identity formation, independence, and autonomy. From this perspective, differences in how parents and adolescents perceive family functioning may reflect a natural process of individuation, which is important for long‐term healthy development (Grotevant & Cooper, 1986; Lerner et al., 2015). Early adolescents want to be independent and self‐directed, whereas parents want to remain close, which leads to an increase in discrepancies between the two (Collins & Laursen, 2004; Steinberg & Morris, 2001). As adolescents move into mid‐to‐late adolescence, their need for autonomy continues to grow, but the rate at which this discrepancy increases begins to slow. During this period, parents often adjust their parenting practices to better meet their adolescents' need for autonomy, which reduces discrepancies between perspectives of the two parties (Collins & Laursen, 2004; Steinberg & Morris, 2001). Also, as they mature, adolescents develop more advanced perspective‐taking abilities. This development enables adolescents to gain a better understanding of their parents' viewpoints, likely leading to a closer alignment in adolescent‐parent perceptions of parents' behaviors (Korelitz & Garber, 2016; Steinberg & Morris, 2001).

Previous meta‐analyses examining cross‐sectional age effects support the normative nature of discrepancy resulting from adaptation processes, showing that parent–adolescent concordance in reports of parental practices tends to be higher among older adolescents compared with younger ones (Hou et al., 2020; Korelitz & Garber, 2016). Longitudinal research is less common but provides pertinent information. For instance, Mastrotheodoros et al. (2020) investigated discrepancies in reports of conflict intensity among parents and adolescents aged 13 to 18. The discrepancy between parent and adolescent reports increased significantly between the ages of 13 and 15, with adolescents consistently reporting higher levels of conflict intensity compared with their parents. Gniewosz et al. (2023) examined discrepancies in perceptions of parental warmth with child participants who were around 8 years old at the beginning of the study and followed for 4 years. In mother–child dyads, the ratings from both informants seemed consistent until the children reached about 10 years of age; significant discrepancies began to appear 1 year later. In contrast, for father–child dyads, differences between the informants' reports were relatively small but significant at all ages. In general, these findings suggest that early adolescence is a critical period for understanding the dynamics of discrepancy between parents and adolescents.

### Parent–adolescent discrepancies and internalizing symptoms

In adolescence, emerging psychological issues, particularly internalizing symptoms, can significantly impact developmental trajectories and shape long‐term social–emotional functioning and overall well‐being (Danneel et al., 2019; Liu et al., 2023). In this context, parent–adolescent discrepancies in perceptions of parenting practices play a crucial role as these misalignments can take distinct forms, each linked to specific psychological outcomes in adolescents (De Los Reyes & Ohannessian, 2016).

When adolescents report higher levels of positive parenting or closeness than their parents, they tend to experience better adjustment outcomes. For example, Mexican‐origin adolescents who scored higher than their parents on parenting measures reported fewer physical functioning problems compared with adolescents whose scores were similar to or lower than their parents (Hou et al., 2018). In contrast, when parents report higher levels of positive parenting—or lower levels of negative parenting—than their adolescents, such discrepancies may indicate underlying family functioning issues that can contribute to adolescents' psychosocial maladjustment (De Los Reyes et al., 2016; Ohannessian & De Los Reyes, 2014).

The maladaptive model offers an alternative interpretation of parent–adolescent discrepancy in perceptions of parenting constructs (De Los Reyes & Ohannessian, 2016). It suggests that discrepancies arise from dysfunctional or maladaptive family dynamics, with stress and disorganization serving as key contributing factors. This situation particularly arises during early adolescence and in high‐risk families. When parents lack insight into family issues or their adolescents' perceptions, adolescents are less likely to receive essential support for navigating negative family environments, whether real or perceived (De Los Reyes & Ohannessian, 2016).

Empirical evidence from both cross‐sectional and longitudinal studies generally supports the modified operations triad model (De Los Reyes & Ohannessian, 2016), showing that larger parent–adolescent discrepancies correlate with increased internalizing symptoms (Fan et al., 2023; Kapetanovic & Boson, 2022; Nelemans et al., 2016; Nichols & Tanner‐Smith, 2022). Such parent–adolescent discrepancies can heighten emotional distress due to misalignment between adolescents' developmental needs and parents' expectations, particularly during the critical period when adolescents are seeking greater autonomy (Collins & Laursen, 2004; Eccles et al., 1993; Lerner et al., 2015). As a result, adolescents may feel misunderstood, which can contribute to higher levels of internalizing symptoms (De Goede et al., 2009). This hypothesis is also consistent with interpersonal theories of developmental psychopathology, which emphasize the importance of relationships in predicting adolescent adjustment (Rudolph et al., 2016).

A variety of methodological approaches support the view that greater parent–adolescent discrepancies are associated with increased internalizing symptoms. Earlier studies used discrepancy scores (e.g., Guion et al., 2009; Hou et al., 2018), which have been criticized on methodological grounds (Laird & De Los Reyes, 2013). Studies employing polynomial regression analysis have provided partial support for the modified operations triad model, revealing the link between discrepancies and internalizing symptoms only in certain conditions (Nelemans et al., 2016; Zhai et al., 2025). For instance, Nelemans et al. (2016) found a significant longitudinal relation between discrepancies and internalizing symptoms only in father–child dyads, raising questions about the generalizability of the association. Zhai et al. (2025) reported a similar result, indicating that discrepancies in reports of behavioral control, with adolescents scoring lower than their fathers, were associated with higher levels of anxiety in adolescents, but this association was observed only cross‐sectionally.

Other studies using different methodological approaches have challenged the directional association between parent–adolescent discrepancies and adolescent adjustment. Using latent congruence models (Cheung, 2009) with the Dutch RADAR sample, Nelemans et al. (2023) found that the overall mother–adolescent relationship quality, rather than discrepancies in perceptions of parenting constructs, serves as a more robust longitudinal predictor of adolescent depressive and anxiety symptoms. They also observed a longitudinal effect from adolescent internalizing symptoms to mother–adolescent discrepancies in parental control. These results align with alternative theoretical explanations for parent–child discrepancies, such as the depression‐distortion hypothesis (Richters, 1992), which suggests that depressive symptoms may bias individuals' perceptions by increasing the tendency to recall negative information, thus influencing how social interactions are interpreted. Supporting this idea, Rote and Smetana (2016) proposed that perceptual biases—where individuals differently notice, interpret, and recall behaviors—may particularly impact family interaction ratings (De Los Reyes & Kazdin, 2005).

However, empirical evidence remains limited regarding whether youth psychopathological symptoms predict stronger parent–adolescent discrepancies over time. Further complicating this picture, Nelemans et al.'s (2023) findings were specific to the dimension of conflict intensity and did not apply to warmth. Additionally, Vrolijk et al. (2023), analyzing the same Nelemans et al.'s (2023) cohort through a random intercept model to control for between‐family variation, found only cross‐sectional, not longitudinal, associations between parent–child reports of parental autonomy support and child depression. Given the limitations of existing research, more longitudinal studies are needed to clarify the directionality of how youth psychopathological symptoms may contribute to parent–adolescent discrepancies in parental behavior perceptions over time.

### Parent–adolescent discrepancies in parental warmth

To date, only a limited number of studies have explored the impact of discrepancies in reporting about parental warmth on developmental outcomes, particularly in relation to adolescent socioemotional development (Gniewosz et al., 2023; Nelemans et al., 2023; Sloan et al., 2025; Wen et al., 2023), and they have provided only mixed results. In a longitudinal study spanning 4 years, from Grade 3 to Grade 6, Gniewosz et al. (2023) found that the mother–child discrepancy in parental warmth reports solely at Grade 5 (when the children were approximately 10 years old) predicted changes in emotional problems one year later, with mothers' overrating of parental warmth predicting poorer psychological adjustment over time (i.e., a smaller reduction in emotional problems). In father–child dyads, discrepancies in perceived parental warmth were associated with changes in emotional problems at all time points.

Using a person‐centered approach, Sloan et al. (2025) found no significant associations between profiles of parent–adolescent discrepancy over parental warmth and anxiety and depression 3 years later. Similarly, Nelemans et al. (2023) investigated the longitudinal and hypothetically bidirectional relation between discrepancies in mothers' and adolescents' perceptions of parental warmth and adolescent internalizing symptoms. Their findings only revealed unidirectional effects, with lower overall levels of warmth significantly predicting higher depressive symptoms in adolescents 1 year later; discrepancies between mothers' and adolescents' perceptions of warmth did not predict changes in internalizing symptoms over time, nor did internalizing symptoms predict future discrepancies. Taken together, these findings underscore the need for studies that specifically focus on the role of discrepancies in parent–adolescent reports about parental warmth and their developmental implications, particularly through longitudinal designs that target‐specific age ranges during adolescence—when shifts in autonomy and cognitive development may heighten the relevance of such discrepancies—and include populations beyond Western contexts, where family expectations may differ.

### The present study

Building on previous research that has investigated parent–adolescent discrepancies in perceptions of relationship quality and their associations with adolescent adjustment, the present study extends the literature by adopting the interpersonal acceptance–rejection theory (IPARTheory; Rohner, 2004, 2021) as a theoretical framework to examine these discrepancies across cultural contexts. IPARTheory emphasizes the subjective perceptions of parental behaviors held by children rather than parental behaviors themselves (Rohner, 2004), which makes it suitable for studying discrepancies in parent–child perceptions.

Also, IPARTheory recognizes that parental warmth can be expressed through several forms, including physical gestures (e.g., hugging, kissing, caressing), verbal affirmations (e.g., praising, complimenting), and symbolic actions informed by specific cultural practices. This understanding of parental warmth as primarily symbolic makes it especially relevant for cross‐cultural research. Specifically, the theory's cross‐cultural applicability is reflected in the development of the Parental Acceptance–Rejection Questionnaire (PARQ; Rohner & Khaleque, 2005), which uses culturally neutral language and common international referents that transcend idiomatic expressions. This measurement approach allows for meaningful comparisons across different cultural contexts while maintaining sensitivity to cultural variations in how parental warmth is expressed and perceived. Despite its importance, the literature has not thoroughly examined cross‐cultural effects on parent–adolescent discrepancies. Although earlier research on cross‐informant discrepancies in adolescent mental health consistently underscored the potential significance of a cultural perspective (Rescorla et al., 2013), this focus has been largely neglected in studies of parent–adolescent discrepancies in reports of family functioning (Rescorla et al., 2017). Increasingly, however, researchers are calling for the inclusion of cross‐cultural comparisons to enhance our understanding of cross‐informant discrepancies (Rescorla et al., 2017).

Parental expressions of warmth are associated with fewer adolescent internalizing problems (Rothenberg et al., 2020, 2022) and better outcomes in several cultures (Khaleque & Ali, 2017). Using the same multinational sample as the present study, Rothenberg et al. (2020) found that warmth protected against the emergence or growth of internalizing symptoms between ages 8 and 14 in nearly all participating cultural groups. Extending this work, Rothenberg et al. (2022) showed that higher levels of parental warmth were consistently associated with fewer internalizing problems, underscoring that variation in parental warmth remains a powerful correlate of child adjustment, regardless of baseline differences in warmth and internalizing levels across cultures and cultural settings. Also, research grounded in this theory shows that children's and adolescents' perceptions of their parents' warmth are more strongly linked to their overall well‐being than the specific behaviors parents use to convey that warmth (Rohner & Lansford, 2017). Supporting the importance of subjective perceptions, Jager et al. (2016) found that early adolescents' unique perspective of parental acceptance–rejection was more strongly associated with adjustment outcomes than shared parent–adolescent perceptions. Despite these advances, no studies have systematically applied IPARTheory to investigate parent–adolescent discrepancies in warmth and across multiple cultural contexts.

The present study aims to examine parent–adolescent discrepancies in perceived maternal and paternal warmth, investigating both their bidirectional associations with adolescent internalizing problems and cross‐cultural variations in the magnitude of these discrepancies. Specifically, we address three main research questions: (1) To what extent do discrepancies in perceptions of parental warmth emerge between adolescents and their mothers and fathers during early to mid‐adolescence (ages 11–15)? (2) Are these discrepancies bidirectionally associated with adolescent internalizing problems over time—that is, do they predict, or are they predicted by, internalizing symptoms? (3) Does the magnitude of these discrepancies vary across cultures? To this end, we applied latent congruence modeling (LCM; Cheung, 2009), which allows for the simultaneous examination of both mean levels and discrepancies in parent and adolescent perceptions. We hypothesized that significant discrepancies would emerge, with parents reporting higher levels of warmth in the relationship compared with adolescents. This expectation is grounded in the unique characteristics of this developmental stage, which includes key normative processes and transitional experiences that may intensify differences in how parents and adolescents perceive warmth in their relationships (De Los Reyes et al., 2019; De Los Reyes & Kazdin, 2005). Regarding the relation between these discrepancies and internalizing symptoms, we did not propose specific hypotheses due to the mixed findings reported in the existing literature. Similarly, we refrained from formulating hypotheses about the variations across cultural contexts, acknowledging the complexity and variability across cultural backgrounds.

### The conceptualization of culture in the current study

Consistent with cultural approaches to parenting research (Bornstein, 2012), culture is conceptualized in this study as the set of distinctive patterns of beliefs and behaviors that are shared by a group of people and that serve to regulate their daily living. From this perspective, culture represents a dynamic system in which cultural beliefs shape parenting cognitions, which in turn influence parenting practices that ultimately prepare children to become culturally competent members of their society (Bornstein, 2012). To operationalize this theoretical framework, the countries included in this research—China, Colombia, Italy, Jordan, Kenya, Philippines, Sweden, Thailand, and the United States—were strategically selected to maximize variation across multiple theoretically relevant cultural dimensions identified by Hofstede (2001), moving beyond abstract conceptualizations to provide concrete, measurable distinctions between cultural groups in family functioning and parent–adolescent dynamics (Lansford et al., 2021). In terms of power distance, Sweden and the United States score relatively low, with families encouraging child independence and the questioning of authority; Colombia, Italy, Jordan, Kenya, and Thailand fall in the moderate range; and China and the Philippines score high, where parent–child relationships emphasize respect, obedience, and hierarchical order. Uncertainty avoidance shows a similar spectrum. In countries such as Sweden and the United States, families tend to display low uncertainty avoidance, characterized by flexible rules and a tolerance for ambiguity. By contrast, Italy and Colombia exemplify high uncertainty avoidance, where families enforce stricter routines and show stronger emotional responses to rule violations. Individualism versus collectivism also reveals important insights. At the collectivist end, countries such as Kenya, Colombia, the Philippines, Thailand, and Jordan emphasize extended family networks and loyalty to the group. China and Italy occupy an intermediate position, balancing group belonging with individual goals. Sweden and the United States represent the individualist end, where nuclear family independence and personal autonomy are prioritized. Long‐term orientation varies as well. Families in China and Thailand score high, valuing perseverance and self‐reliance, whereas Colombia and Kenya lean toward shorter‐term orientations that emphasize tolerance, respect for traditions, and interpersonal harmony. Finally, the dimension of motivation toward achievement and success (formerly referred to as masculinity) distinguishes societies such as China, Colombia, Italy, and the United States, which emphasize competition and success, from Sweden, where families tend to favor more cooperative and nurturing values.

Beyond these differences in cultural values, the participating countries also demonstrate remarkable diversity across sociodemographic indicators (ethnicity, religion, economic status), ranging from 8 to 147 on the Human Development Index, with corresponding disparities in child welfare outcomes, such as infant mortality rates that vary fortyfold across nations (Lansford et al., 2021). Importantly, however, cultural variation is not confined to differences between nations. Meaningful diversity also exists within countries, shaped by historical, ethnic, regional, and socioeconomic factors. For example, in Italy, Rome and Naples were selected to represent the documented North–South cultural divide, particularly in relation to family collectivism, authority relationships, and socioeconomic contexts that influence parenting practices (Bacchini et al., 2024). In the United States, White, Black, and Latino families were examined as distinct cultural groups, based on extensive research that documents systematic differences in parenting values, practices, and orientations persisting beyond individual socioeconomic differences. This within‐country sampling approach makes it possible to capture cultural variation that might otherwise be hidden when entire nations are treated as homogeneous cultural units. This systematic sampling across economic, psychological, and social dimensions makes the project one of the few to investigate which parenting practices reflect universal human patterns and which represent culture‐specific adaptations, while also testing the generalizability of theories developed primarily with Western populations.

## METHOD

### Participants and procedure

Participants included 1219 parent–child dyads (50.6% female children) from the Parenting Across Cultures (PAC) study, a longitudinal investigation of parenting and child development across cultures. As mentioned in the introduction, the nine nations represented are China, Colombia, Italy (with two cultural groups), Jordan, Kenya, the Philippines, Sweden, Thailand, and the United States (with three cultural groups).

The present study used data collected over five years, with measurements taken at three points, each two years apart. The sample consisted of 1150 families with both mother and father participation, 67 mother–child‐only dyads, and 2 father–child‐only dyads. Children's mean age was 10.72 years (*SD* = 0.67) at Wave 1, 13.19 years (*SD* = 0.90) at Wave 2, and 15.60 years (*SD* = 0.94) at Wave 3. Mothers' mean age was 39.33 years (*SD* = 6.51), and fathers' mean age was 42.35 years (*SD* = 6.79) at Wave 1. Most parents (96.2%) were biological parents, and 10.1% were divorced or separated.

The analytic sample for the present study includes families from all 12 cultural groups: Shanghai, China (*n* = 103); Medellín, Colombia (*n* = 100); Naples (*n* = 99); and Rome (*n* = 106), Italy; Zarqa, Jordan (*n* = 113); Kisumu, Kenya (*n* = 99); Manila, Philippines (*n* = 105); Trollhättan/Vänersborg, Sweden (*n* = 102); Chiang Mai, Thailand (*n* = 110); and Durham, NC, United States (*n* = 102 White, *n* = 94 Black, *n* = 86 Latino). Participants were recruited through letters sent to schools. Measures were administered in the primary language of each country following forward‐ and back‐translation procedures to ensure conceptual equivalence (Brislin, 1970). Interviews lasting 1.5–2 h were conducted at each wave after obtaining parent consent and child assent.

Socioeconomic status was sampled in proportions representative of each recruitment area, resulting in an economically diverse sample that ranged from low to high income within each site. Demographic information about family income, parental employment, and religion across countries is reported in Table 1. Child age and gender did not vary across countries. Attrition was minimal: 92% and 83% of the original sample provided data at the second and third data collection points, respectively. Participants who provided follow‐up data did not differ from the original sample with respect to any study variables (Little's test = 441.493, *df* = 422, *p* = .093).

**TABLE 1:** Demographic characteristics by cultural group (valid percentages).

	China	Italy‐Naples	Italy–Rome	Kenya	Philippines	Thailand	Sweden	U.S. Black	U.S. White	U.S. Latino	Colombia	Jordan
*Employment*												
Mothers employed	79.6%	42.1%	78.8%	83.2%	65%	85.1%	95.7%	65.2%	76.2%	61%	50%	24.1%
Fathers employed	93.4%	94.6%	92.6%	93.6%	85.1%	98.9%	98.8%	83%	96.6%	89.7%	92.9%	92.6%
*Religion*												
Catholic	1.1%	95.8%	80.8%	27.4%	85.4%	–	2.4%	1.1%	15.0%	59.5%	94.0%	–
Protestant	5.7%	–	1.0%	67.4%	4.9%	1.0%	56.0%	72.0%	52.0%	25.3%	2.0%	–
Jewish	–	–	–	–	–	–	–	–	5.0%	–	–	–
Buddhist	12.6%	1.1%	1.0%	–	–	99.0%	–	–	1.0%	1.3%	–	–
Hindu	–	–	–	–	–	–	–	–	–	–	–	–
Muslim	–	–	1.0%			–	–	1.1%	–	–	–	100.0%
Other	–	–	2.0%	5.3%	8.7%	–	6.0%	15.1%	12.0%	2.5%	4.0%	–
No religious affiliation	80.5%	3.2%	14.1%	–	1.0%	–	35.7%	10.8%	15.0%	11.4%	–	–

### Measures

#### Demographics

Child gender (coded as 1 for male and 2 for female) and the number of years of parents' education were included in the analyses as covariates.

#### Parental warmth

Parental warmth, as reported by the adolescent and the parent, was assessed using the Parental Acceptance–Rejection Questionnaire‐Short Form (PARQ‐SF; Rohner & Khaleque, 2005). The PARQ‐SF is a widely validated instrument that has demonstrated excellent psychometric properties, including strong reliability, convergent and discriminant validity, and measurement invariance across cultural contexts. This measure has been successfully implemented in more than 60 cultures worldwide (Rohner & Khaleque, 2005), and it has been extensively used by the PAC research team and others across all nine participating countries in the present study (Lansford et al., 2018). Furthermore, previous PAC investigations utilizing the alignment method (Asparouhov & Muthén, 2014) have found consistent relative invariance across all cultures at all time points (Lansford et al., 2021).

To maintain model parsimony, for the present study, we selected four key items that theoretically align with Rohner's (2021) conceptualization of parental acceptance and specifically capture parental warmth: expressing affection (“says nice things to the child”), facilitating open communication (“makes it easy to confide”), conveying significance (“makes the child feel important”), and demonstrating emotional support (“cares about and encourages child's thoughts”). This selection captures both the behavioral manifestations and emotional climate that IPARTheory identifies as central to the warmth dimension, ensuring theoretical coherence across cultures. Both parents and adolescents rated the frequency of these behaviors on a 4‐point scale ranging from 1 (*almost never*) to 4 (*every day*), with higher scores indicating greater parental warmth. The measure was assessed at Wave 1 and Wave 2. Cronbach alphas and McDonald's omegas were between .69 and .70 for parents' reports and between .70 and .75 for adolescents' reports.

#### Internalizing symptoms

Adolescent internalizing symptoms were assessed using the Internalizing Behavior scale from the Youth Self‐Report (Achenbach & Rescorla, 2001), which includes 29 items measuring various emotional and behavioral issues such as anxiety and depression, withdrawn behavior, and somatic complaints. Adolescents rated how true each item was of their experience during the previous 6 months using a 3‐point scale (0 = *not true*, 1 = *somewhat or sometimes true*, 2 = *very or often true*). The measure was assessed at all time points. This measure has demonstrated validity and reliability across all cultural groups in the present study (e.g., Lansford et al., 2018) and has been successfully employed in these cultures by our own and other research groups (e.g., Deater‐Deckard et al., 2018; Rescorla et al., 2007). Indices of internal consistency in this study were adequate across all assessment waves, with Cronbach's alpha coefficients ranging from .73 to .87 and McDonald's omegas from .74 to .87.

### Analytic plan

The analytic plan involved a series of steps aimed at investigating parent–adolescent discrepancies in perceptions of parental warmth using latent congruence models (LCMs; Cheung, 2009). Mother–adolescent and father–adolescent dyads were analyzed separately.

First, we tested cross‐informant measurement invariance in perceptions of parental warmth longitudinally, applying the same parameter constraints across time points. Following best practices in the use of LCMs, we tested for configural, metric, and scalar invariance. However, in line with De Los Reyes et al. (2022), we recognize the importance of balancing the application of measurement invariance techniques with the potential consequences for measurement validity. Specifically, De Los Reyes et al. (2022) challenged the indiscriminate application of these techniques, claiming that measurement invariance may not always be required when discrepancies between informants are relevant to the specific context. Specifically, if discrepancies reflect meaningful differences in how parents and adolescents perceive parental warmth, then constraining parameters to be equal may obscure valuable context‐specific variability that is clinically relevant.

In this light, we adopted a flexible approach to measurement invariance, comparing configural, metric, and scalar invariance while allowing parameters to vary when there was evidence for domain‐relevant meaningful differences. This methodology is consistent with the “Measurement Invariance Oath” (De Los Reyes et al., 2022), which emphasizes preserving measurement validity and important sources of informational variance by avoiding unnecessary constraints. By allowing for parameter variability when needed, we were able to capture the nuances of parent–adolescent discrepancies while ensuring that our model remained informative and valid for both research and clinical application (Keeley et al., 2024).

Configural invariance was tested by estimating a baseline model without equality constraints across informants, which served as a reference model for subsequent comparisons. Next, we tested metric invariance by constraining the factor loadings to be equal across parents (mother or father) and adolescents. This step assessed whether both informants attribute the same weight to the items in the scale. Finally, scalar invariance was tested by constraining the item intercepts to be equal to determine whether the response categories are interpreted in the same way by both informants in the dyad (adolescent and mother/father). Each step was evaluated by examining the overall fit of the model, including the Chi‐square statistic, root mean square error of approximation (RMSEA), comparative fit index (CFI), and standardized root mean square residual (SRMR), with an acceptable deterioration in fit typically being defined as a ΔCFI < .01 and ΔRMSEA < .015 (Chen, 2007; Cheung & Rensvold, 2002). Given the limitations of the Chi‐square statistic with large sample sizes, we prioritized alternative fit indices with accepted thresholds: RMSEA ≤ .08, CFI ≥ .90, and SRMR ≤ .08 (Kline, 2016). If the metric or scalar invariance model did not fit well, we proceeded with partial invariance by releasing constraints on specific items where needed (Vandenberg & Lance, 2000). After establishing measurement invariance, we employed LCM to investigate mean discrepancies in parents' and adolescents' perceptions of parental warmth using structural equation modeling (SEM). A key advantage of using LCM is that it allows researchers to examine both the antecedents and consequences of informant discrepancies over time, over and above concurrent associations and temporal stability in constructs. Model adequacy was assessed using the same fit indices mentioned above. The discrepancy between these perceptions is modeled as a latent average difference between parents and adolescents (Congruence), capturing the deviation of parent‐reported warmth from adolescent‐reported warmth while controlling for measurement error. In simpler terms, this congruence factor represents the discrepancy in perceived warmth between parents and adolescents. Positive values of the discrepancy score reflect parents reporting higher perceptions of parental warmth than adolescents; negative values indicate that adolescents report higher levels of parental warmth than parents. Additionally, the model includes a latent level factor (Level), which represents the shared variance between parent and adolescent perceptions of parental warmth, reflecting the overall perceived parental warmth in the dyad. We examined whether mean discrepancies in parental warmth are statistically significant, indicating meaningful differences in perceptions of parental warmth between parents and adolescents. Finally, we assessed whether these discrepancies in perceptions of parental warmth between parents and adolescents change over time, providing insights into potential developmental shifts in parent–adolescent discrepancy of parental warmth.

Subsequently, we extended the model to examine longitudinal associations with internalizing symptoms, incorporating this variable as both a predictor and an outcome. Specifically, the analysis investigated whether discrepancies in warmth are predicted by or predict changes in internalizing symptoms over time, over and above the overall level of perceived warmth in the dyad. To account for stability in the constructs over time, we included autoregressive paths for internalizing symptoms as well as for both the level and discrepancy factors. Additionally, we tested a model with equality constraints on the paths linking level and discrepancy scores to internalizing symptoms over time and compared it against the model with these paths freely estimated. Scatterplots are provided as Supplemental Material to display significant associations involving discrepancy scores and facilitate a clearer understanding of the relation (Figure S1a–e). Also, we controlled for relevant covariates of parents' education levels and child gender. Model fit and comparisons between the models were reassessed using the same criteria mentioned earlier.

For the cross‐cultural aspect of the analysis, we examined differences in both the mean level of perceived parental warmth and the discrepancies between parents and adolescents by modeling cultural groups as predictors of the level and discrepancy factors. Cultural groups were coded using deviation coding, which allows each group to be compared with the overall mean rather than to a single reference group. This approach provides estimates of how each cultural group's mean level of perceived parental warmth and discrepancy scores deviate from the grand mean (i.e., the average across all groups), ensuring a more balanced interpretation. Specifically, positive coefficients indicate that a cultural group's score is greater than the overall mean, whereas negative coefficients suggest that it is lower. This method avoids the limitations of traditional dummy coding, where results depend on the choice of a reference category, and instead provides a comprehensive view of cultural variations in warmth perceptions and discrepancies. To ensure parsimony in the estimation of cultural effects over time, we constrained these effects to be equal across time points.

## RESULTS

### Descriptive statistics

The descriptive statistics and correlations for the study variables are presented in Table 2. Adolescent girls increased in internalizing symptoms over time (*r* = .07, *p* < .05 to *r* = .27, *p* < .001). Maternal and paternal education levels were highly correlated (*r* = .71, *p* < .001) and showed minimal associations with other variables. Maternal education, specifically, was positively associated with mother‐reported warmth (*r*s = .10, *p* < .01, and .092, *p* < .05). Internalizing symptoms demonstrated moderate stability across time points (*r* = .36 to *r* = .56, *p*s < .001) and were negatively associated with adolescent‐reported parental warmth for both mothers (*r* = −.06, *p* < .05 to −.21, *p* < .001) and fathers (*r* = −.14 to −.26, *p* < .001) across time. Adolescent reports of maternal and paternal warmth were moderately correlated (*r*s = .61 and .56, *p* < .001, at Wave 1 and Wave 2, respectively). Within‐dyad correlations between parent and adolescent reports of parental warmth were modest for both mothers (*r*s = .36 and .28, *p* < .001) and fathers (*r* = .29 and .27, *p* < .001), suggesting some discrepancy in perceptions of parental warmth.

**TABLE 2:** Means, standard deviations, and correlations.

Variable	*M*	*SD*	1	2	3	4	5	6	7	8	9	10	11	12	13
1. Adolescent Gender	–	–	1												
2. Mother's education (years)	12.72	4.21	−.02	1											
3. Father's education (years)	12.84	4.23	.00	.71***	1										
4. Internalizing symptoms W1	11.74	7.79	.07*	−.00	−.04	1									
5. Internalizing symptoms W2	12.90	8.65	.13***	.00	.01	.46***	1								
6. Internalizing symptoms W3	14.48	9.55	.27***	.00	.00	.36***	.56***	1							
*Parental warmth W1*															
7. Adolescent → Mother	3.53	0.54	.04	.16***	.11***	−.14***	−.06*	−.05	1						
8. Adolescent → Father	3.43	0.63	−.04	.14***	.08**	−.17***	−.14**	−.15***	.61***	1					
9. Mother → Adolescent	3.61	0.48	−.02	.10***	.04	.05	−.02	.02	.36***	.26***	1				
10. Father → Adolescent	3.44	0.55	−.03	.09**	.06	−.06	−.08*	−.08*	.26***	.29***	.38***	1			
*Parental warmth W2*															
11. Adolescent → Mother	3.49	0.59	−.00	.05	.04	−.13***	−.21***	−.17***	.33***	.24***	.20***	.13***	1		
12. Adolescent → Father	3.37	0.66	−.06*	−.01	.04	−.14***	−.26***	−.22***	.24***	.31***	.16***	.21***	.56***	1	
13. Mother → Adolescent	3.59	0.51	−.01	.09**	.07*	.02	−.06	−.08*	.20***	.15***	.42***	.18***	.28***	.24***	1
14. Father → Adolescent	3.45	0.55	−.01	−.00	−.01	−.06	−.12***	−.06	.15***	.14***	.19***	.38***	.21***	.27***	.25***

*Note*: *M* and *SD* represent mean and standard deviation, respectively.

* *p* < .05.

** *p* < .01.

*** *p* < .001.

### Mother–adolescent discrepancy in reports of parental warmth

#### Cross‐informant invariance

To examine cross‐informant measurement invariance across the two waves of the study in which parental warmth was assessed, we tested configural, metric, and scalar invariance while maintaining equality constraints over time. The configural model, which allowed factor loadings and item intercepts to vary, demonstrated good fit (*χ* ^2^(74) = 180.463, *p* < .001; RMSEA = .034, 90% CI [.028, .041]; CFI = .969; SRMR = .036). Next, we tested metric invariance by constraining factor loadings to be equal across informants, while maintaining the same constraints across waves. The initial metric model showed a slight deterioration in CFI (ΔCFI = .016; ΔRMSEA = .005; Δ*χ* ^2^(12) = 64.744, *p* < .001). After releasing the factor loading constraints for the item “Care and encourage thoughts,” the partial metric model demonstrated improved fit (ΔCFI = .01; ΔRMSEA = .003; Δ*χ* ^2^(11) = 43.193, *p* < .001; *χ* ^2^(85) = 223.656, *p* < .001; RMSEA = .037, 90% CI [.031, .042]; CFI = .959; SRMR = .054), indicating that mothers and their adolescents weighted this item differently when considering how much it reflected maternal warmth. This difference may reflect a different emphasis on cognitive‐emotional support, with mothers and adolescents placing different importance on parental validation during identity development. We then tested scalar invariance by constraining item intercepts to be equal across informants, while keeping these constraints across waves. The initial scalar model showed substantial fit deterioration compared with the partial metric model (ΔCFI = .031; ΔRMSEA = .008; Δ*χ* ^2^(12) = 138.553, *p* < .001). After releasing all intercept constraints except for the “Feel important” item, the revised scalar model demonstrated a notably improved fit (*χ* ^2^(94) = 247.026, *p* < .001; RMSEA = .037, 90% CI [.031, .042]; CFI = .955; SRMR = .060), with a significant change in fit (ΔCFI = .004; ΔRMSEA = .000; Δ*χ* ^2^(9) = 23.370, *p* < .01). Specifically, the intercepts for mothers' reports of “easy to confide” and “Care and encourage thoughts” were higher compared with adolescents, potentially reflecting mothers' greater awareness of their emotional availability intentions, whereas “saying nice things” was rated higher by adolescents than by mothers, likely because adolescents are particularly sensitive to explicit verbal affirmations during this developmental period.

#### Latent congruence models

The latent congruence model showed good fit (*χ* ^2^(89) = 226.973, *p* < .001; RMSEA = .036, 90% CI [.036, .041]; CFI = .959; SRMR = .047). Constraining discrepancy scores to be equal across time did not significantly change model fit (ΔCFI = .001; ΔRMSEA = .001; Δ*χ* ^2^ (1) = 0.351, *p* > .05), suggesting that the magnitude of discrepancies between mothers and adolescents was stable across waves. Results revealed a significant mean discrepancy in parental warmth reports between mothers and adolescents (0.036, *p* < .05), indicating that, on average, adolescents perceived lower warmth compared with mothers' reports. The variances at Wave 1 = 0.111 and Wave 2 = 0.164 (*p* < .001) show that dyads differed in the magnitude of their discrepancy scores. Variances of the overall levels of warmth in the dyad were 0.084 and 0.092 (*p* < .001) at W1 and W2, respectively, reflecting significant between‐dyad differences in the general level of warmth experienced.

#### Longitudinal associations with internalizing symptoms

**FIGURE 1:**
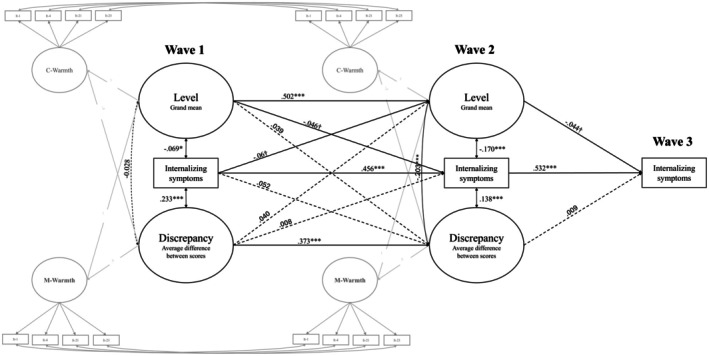
Cross‐lagged and latent congruence model of mother–adolescent warmth discrepancies and adolescent internalizing symptoms. Covariances between mothers' and children's warmth reports within the same wave are omitted in the figure. Standardized coefficients are reported. ***p < .001, **p < .01, *p < .05, †*p* < .10.

The results of the model are presented in Figure 1. The model demonstrated good fit (*χ* ^2^(154) = 329.652, *p* < .001; RMSEA = .031, 90% CI [.026, .035]; CFI = .958; SRMR = .040). Constraining the paths linking level and discrepancy scores to internalizing symptoms over time did not worsen the model fit (ΔCFI = .000; ΔRMSEA = .001; Δ*χ* ^2^ (2) = 2.622, *p* > .05).

Significant autoregressive associations emerged for overall levels of warmth in the dyad, discrepancy scores, and internalizing symptoms. No significant cross‐lagged associations were found for level and discrepancy scores. Adolescent internalizing symptoms at Wave 1 were marginally and negatively associated with the level of maternal warmth 2.5 years later (Wave 2); no association was found between internalizing symptoms at Wave 1 and mother–adolescent discrepancy at Wave 2. Additionally, a marginally significant association was observed between levels of parental warmth at Wave 1 and Wave 2 and subsequent internalizing symptoms (Wave 2 and Wave 3, respectively), after controlling for temporal stability. That is, higher overall levels of warmth were associated with decreasing internalizing symptoms over time. Such an association was not found for the discrepancy scores.

Cross‐sectionally, at Wave 2, we observed an inverse relation between dyadic warmth levels and discrepancy scores. The inspection of the scatterplot (Figure S1a) revealed that higher warmth levels in mother–adolescent dyads were associated with decreased positive discrepancy scores (i.e., decreased overrating of mothers compared with adolescents). At both Wave 1 and Wave 2, warmth levels and discrepancy scores were significantly associated with adolescent internalizing symptoms. Higher overall dyadic warmth was associated with fewer internalizing symptoms. Notably, the direction of discrepancies in warmth perceptions mattered: when adolescents reported higher warmth than their mothers (negative discrepancy), internalizing symptoms were lower; conversely, when mothers reported higher warmth than adolescents perceived (positive discrepancy), adolescents showed increased internalizing symptomatology (Figure S1b).

Regarding control variables, maternal education was positively associated with overall warmth levels in the dyad at both waves (*β*s = .169 and .056, *p*s < .001 and <.05, respectively) but not with discrepancy (*β* = −0.024 and −0.007, *p*s = .502 and .832, respectively). At both waves, child gender was unrelated to warmth levels (*β* = 0.001 and 0.024, *p*s = .969 and .365, respectively) or discrepancy (*β*s = −0.020 and −0.050, *p*s = .583 and .140, respectively).

#### Cross‐cultural effects

The analysis revealed significant cultural group effects on the mean level of perceived warmth in mother–adolescent dyads. Specifically, dyads in China, Kenya, Thailand, and Jordan reported lower warmth levels compared with the group mean, whereas dyads in Naples, Sweden, Colombia, and the United States (Black and White) reported higher levels. Cultural group differences in parent–adolescent warmth discrepancy were minimal, with only dyads in the Philippines showing a significantly higher discrepancy (*p* = .001) and white U.S. Americans having a lower discrepancy score (*p* = .042) compared with the group mean. In addition to the cross‐cultural effects described here, descriptive statistics for each cultural group (i.e., mean levels of perceived warmth, discrepancies, and internalizing symptoms) are presented in Supplemental Material (Table S1).

### Father–adolescent discrepancy

#### Cross‐informant invariance

The configural model, testing the equality of factor structure, demonstrated adequate fit (*χ* ^2^(74) = 202.640, *p* = .001; RMSEA = .039, 90% CI [.033, .045]; CFI = .963; SRMR = .034). Testing for metric invariance by constraining factor loadings across informants resulted in a significant decrease in CFI (ΔCFI = .014; ΔRMSEA = −.003; Δ*χ* ^2^(12) = 60.439, *p* < .001). As in the mother model, we achieved partial metric invariance by releasing the constraint for the item “Care and encourage thoughts,” which significantly improved model fit (ΔCFI = .008; ΔRMSEA = −.001; Δ*χ* ^2^(11) = 39.545, *p* < .001; *χ* ^2^(85) = 242.185, *p* < .001; RMSEA = .040, 90% CI [.034, .046]; CFI = .955; SRMR = .054). The full scalar invariance model, constraining item intercepts to equality, showed poor fit (ΔCFI = .041; ΔRMSEA = −.012; Δ*χ* ^2^(12) = 152.017, p < .001). However, model fit was improved by retaining the intercept constraint only for the “Feel important” item (*χ* ^2^(94) = 275.295, *p* < .001; RMSEA = .041, 90% CI [.035, .047]; CFI = .948; SRMR = .057; ΔCFI = .007; ΔRMSEA = −.001; Δ*χ* ^2^(9) = 33.110, *p* < .001). These results indicate that although fathers and adolescents share some similarities in how they conceptualize parental warmth, they show substantial differences in their response patterns across most items. As in the mother model, the intercepts for fathers' reports of “easy to confide” and “Care and encourage thoughts” were higher compared with adolescents, whereas “saying nice things” was rated higher by adolescents than by fathers.

#### Latent Congruence Models

The latent congruence model for father–adolescent reports demonstrated good fit to the data (*χ* ^2^(89) = 250.797, *p* = .001; RMSEA = .040, 90% CI [.034, .046]; CFI = .953; SRMR = .042). Constraining discrepancy scores to be equal across time did not significantly change model fit (ΔCFI = .001; ΔRMSEA = .000; Δ*χ* ^2^ (1) = 5.16, *p* < .05), suggesting that the magnitude of discrepancies between fathers and adolescents was stable across waves. The results revealed a negative mean discrepancy in reports of parental warmth between fathers and adolescents (i.e., adolescents reporting higher warmth compared with fathers), which was not significant (−0.018, *p* = .364). Variances of the discrepancy score at Wave 1 were .238 and .233 at Wave 2 (*p* < .001), indicating that dyads differed in the magnitude of their discrepancy scores. The variances of the overall levels of warmth in the dyad were .119 and .126 (*p* < .001) at Wave 1 and Wave 2, respectively, reflecting significant between‐dyad differences in the general level of warmth experienced.

#### Longitudinal associations with internalizing symptoms

**FIGURE 2:**
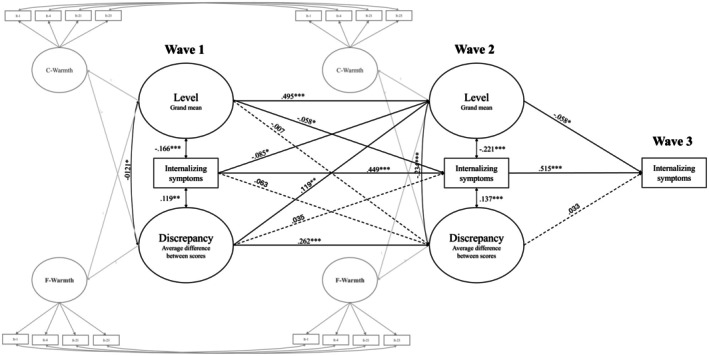
Cross‐lagged and latent congruence model of father–adolescent warmth discrepancies and adolescent internalizing symptoms. Covariances between fathers' and children's warmth reports within the same wave are omitted in the figure. Standardized coefficients are reported. ***p < .001, **p < .01, *p < .05.

The results of the model including internalizing symptoms are displayed in Figure 2. The model demonstrated good fit to the data (*χ* ^2^(154) = 369.618, *p* < .001; RMSEA = .035, 90% CI [.030, .039]; CFI = .949; SRMR = .038). Constraining the paths linking level and discrepancy scores to internalizing symptoms over time did not worsen the model fit (ΔCFI = .000; ΔRMSEA = .000; Δ*χ* ^2^ (2) = 3.073, *p* > .05).

Internalizing symptoms and both overall warmth levels and discrepancy scores demonstrated significant autoregressive associations across waves. Furthermore, we observed a positive cross‐lagged relation between Wave 1 Discrepancy and Wave 2 Level. Specifically, as the scatterplot revealed (Figure S1c), the predicted Wave 2 Level becomes increasingly positive as the discrepancy approaches zero (i.e., fathers' and adolescents' perceptions match) or becomes more positive (i.e., fathers report more warmth than adolescents). Additionally, higher levels of adolescent internalizing symptoms two years earlier (Wave 1) were significantly associated with lower mean levels of paternal warmth in the dyad (Wave 2) but not with discrepancy. Finally, mean levels of warmth in the dyad were negatively associated with internalizing symptoms over time, after controlling for temporal stability. Conversely, discrepancy scores showed no significant longitudinal relations with internalizing symptoms.

Cross‐sectionally, at both Wave 1 and Wave 2, higher overall warmth was associated with reduced positive discrepancy scores (operationalized as fathers reporting higher warmth than adolescents; Figure S1d). Both warmth levels and discrepancy factors at Waves 1 and 2 showed significant relations with adolescent internalizing symptoms. Specifically, greater dyadic warmth was associated with fewer internalizing symptoms in adolescents. As in the mother model, the directional nature of discrepancies proved particularly meaningful: adolescents who perceived greater paternal warmth than their fathers themselves reported (negative discrepancy) displayed lower internalizing symptomatology, whereas those whose fathers reported higher warmth than adolescents perceived (positive discrepancy) exhibited increased internalizing symptoms (Figure S1e).

Control variables showed that both fathers' education and adolescent gender were associated with Wave 1 overall warmth levels (*β*s = .082 and − .070, *p*s = .014 and .031, respectively), with dyads with boys reporting higher warmth than those with girls. No significant associations were found neither with overall levels of warmth at Wave 2 (father's education: *β* = .025, *p* = .371; adolescent gender: *β* = .012, *p* = .672) nor with discrepancy scores at both waves (father's education: *β*s = −.027 and − .044, *p*s = .446 and .214, for Wave 1 and Wave 2, respectively; adolescent gender: *β*s = .007 and .038, *p*s = .845 and .283, for Wave 1 and Wave 2, respectively).

#### Cross‐cultural effects

The analysis revealed significant cultural group effects on the mean level of perceived warmth in the father–adolescent relationship. Specifically, dyads in China, Rome, Kenya, Thailand, and Jordan reported significantly lower levels of warmth compared with the overall group mean, whereas dyads in Sweden, the United States (all ethnic groups), and Colombia reported significantly higher levels. Regarding discrepancy scores between father and adolescent perceptions of warmth, most cultural groups did not show significant differences, with four exceptions: Colombia and the Philippines demonstrated significantly higher discrepancy (*β* = .142, *p* = .002; *β* = .103, *p* = .024), whereas Thailand and the White U.S. groups showed significantly lower discrepancy (*β* = −.126 and −.139, *p* = .004) compared with the overall grand mean. In addition to the cross‐cultural effects described here, descriptive statistics for each cultural group (i.e., mean levels of perceived warmth, discrepancies, and internalizing symptoms) are presented in Supplemental Material (Table S2).

## DISCUSSION

Grounded in IPARTheory (Rohner, 2004, 2021), this study contributes to the extant literature on the complex and dynamic nature of perceptions of parental warmth in parent–adolescent relationships (Research Question 1). By analyzing the bidirectional relation between discrepancies in these perceptions and adolescent internalizing symptoms over time, our findings provide insights into how parent–adolescent discrepancies in parental warmth are associated and may shape—and be shaped by—adolescents' psychological adjustment (Research Question 2). Furthermore, the study discusses variations in parent–adolescent discrepancies across different cultural contexts (Research Question 3), further enriching our understanding of these dynamics.

As regards our Research Question 1, the findings revealed distinct patterns in mother–adolescent and father–adolescent relationships. For mothers, we found significant and stable positive discrepancies over time, indicating that mothers consistently reported higher levels of warmth than their adolescents. In contrast, father–adolescent dyads showed no significant discrepancies, although there was a tendency for adolescents to report higher warmth than fathers. Several key patterns linking parent–adolescent discrepancies and adolescent internalizing symptoms emerged (Research Question 2). First, higher levels of parental warmth predicted fewer internalizing symptoms over time, and higher internalizing symptoms predicted lower subsequent warmth levels, suggesting a bidirectional relation. Second, cross‐sectionally in both mother and father models, higher levels of overall warmth in the dyad were associated with smaller positive discrepancy scores and lower internalizing symptoms. When parents reported higher warmth than adolescents perceived (positive discrepancy), adolescents exhibited higher internalizing symptoms. Conversely, when adolescents reported higher warmth than parents (negative discrepancy), internalizing symptoms were lower. Third, despite these concurrent associations, we found no longitudinal relations between discrepancies and internalizing symptoms in either direction. Fourth, a unique pattern emerged in father–adolescent relationships: as the discrepancy score at Wave 1 shifted from negative toward positive values (where fathers reported higher warmth than adolescents), overall warmth levels at Wave 2 progressively increased, suggesting that these perception differences may evolve over time. Finally, addressing Research Question 3, we identified a few cross‐cultural differences in the magnitude of parent–adolescent discrepancies.

### Parent–adolescent discrepancies in parental warmth

The significant positive discrepancies in mother–adolescent dyads—where mothers reported higher warmth than adolescents—align with both the generational stake hypothesis (Bengtson & Kuypers, 1971) and the adaptive perspective of the modified operation triad model (De Los Reyes & Ohannessian, 2016; Ohannessian et al., 2000), which emphasizes developmental differences in family perceptions as largely driven by developmental changes during adolescence (Smetana & Rote, 2019). As adolescents seek greater autonomy and independence, they may perceive less warmth from parents, whereas mothers, invested in relationship continuity, tend to emphasize positive aspects of parenting (Grotevant & Cooper, 1986; Lerner et al., 2015). This finding is also consistent with prior research showing that parents generally report more positive parenting practices than their children (Augenstein et al., 2016; De Los Reyes & Ohannessian, 2016; Gaylord et al., 2003), including warmth (Dotterer & Day, 2019; Hou et al., 2018; Wen et al., 2023).

The pattern observed in the father–adolescent dyads presents an interesting contrast. Although the discrepancies in perceived warmth between fathers and adolescents were not statistically significant in the present study, there was a consistent tendency for adolescents to report slightly higher levels of warmth than their fathers. This trend is consistent with the small but growing body of studies finding that adolescents report more positive perceptions than their parents (Fan et al., 2023; Gniewosz et al., 2023; Rote & Smetana, 2016; Wen et al., 2023). Gniewosz et al. (2023), for instance, found that father–child discrepancies tend to be small but significant across different ages, with adolescents reporting higher levels than their fathers. To explain this pattern, Hou et al. (2018) argued that as adolescents develop autonomy, empathy, and perspective‐taking, consistent with broader developmental trends in adolescent cognition, they may gain a deeper appreciation for parental sacrifices. However, this tendency was observed only in father–adolescent dyads, thus suggesting that alternative interpretations, which consider the different roles of mothers and fathers in parent–adolescent relationships, are necessary. For example, traditional views of parental warmth have primarily been associated with maternal roles and feminine expressions of care (Cabrera et al., 2014; Pleck, 2010). As a result, fathers may underestimate their expressions of warmth when assessing them through these conventional lenses. Additionally, research in IPARTheory indicates that fathers often express warmth in less overtly affectionate ways, such as through shared activities, instrumental support, or behavioral involvement (Rohner & Veneziano, 2001). In this context, adolescents may be particularly receptive to these paternal behaviors and interpret them as expressions of warmth (Lamb, 2010), which could explain their higher reports. This interpretation is supported by previous studies showing that father–child relationships often develop through different pathways than mother–child relationships, with fathers expressing care and connection through unique behavioral patterns that may not align with traditional measures of parental warmth (Cabrera et al., 2014; Paquette, 2004). However, it is important to note that this remains a tentative interpretation, as the observed tendency did not reach statistical significance, which may reflect a subtle pattern that warrants further investigation through more sensitive methodological approaches.

The relation between discrepancies and warmth levels showed different temporal patterns. Cross‐sectional analyses revealed that greater parent–adolescent agreement in perceptions of warmth was associated with higher overall warmth levels and vice versa. This finding is consistent with research suggesting that when parent–adolescent relationships are characterized by high warmth, there is often greater concordance between parents and adolescents (De Los Reyes et al., 2019). Longitudinally, a significant positive relation between parent–adolescent discrepancy and overall levels of warmth emerged only in father–adolescent dyads, where initial discrepancies predicted subsequent warmth levels. Specifically, when father–adolescent perceptions at Wave 1 were more aligned—or when adolescents reported less warmth than their fathers did—perceived overall warmth levels increased 2+ years later. In contrast, dyads in which adolescents initially reported greater warmth than their fathers perceived were characterized by lower overall warmth at Wave 2. Two explanations for this pattern are possible. First, the decline in warmth over time could stem from fathers' underestimation of their nurturing behaviors. When fathers fail to recognize their warmth relative to adolescents' perceptions, they may not intentionally reinforce those positive behaviors, leading to a gradual reduction over time. Second, adolescents who initially perceive greater warmth than their fathers acknowledge may experience a shift in perception due to disappointment or a lack of reciprocal emotional validation. This growing discrepancy could lead to withdrawal or reduced positive engagement from adolescents, ultimately contributing to a decline in overall levels of warmth in the dyad.

When adolescents report lower warmth than their fathers (i.e., a positive discrepancy score at Wave 1), cognitive maturation may play a key role in shaping adolescents' perceptions of parental behaviors over time. As adolescents develop, their ability to understand parental intentions and adopt their parents' perspective improves significantly. This maturation may lead adolescents to a more realistic assessment of paternal warmth, moving away from idealized or overly critical views of early adolescence. Steinberg and Morris (2001) documented that parent–adolescent relationships generally improve as late adolescents develop more sophisticated understandings of their parents' perspectives and behaviors. This cognitive growth could explain why greater alignment of adolescents' perceptions with those of their fathers at Wave 1 predicts increased warmth in the dyad 2 years later. Future studies adopting person‐centered approaches could provide deeper insights and clarity regarding this point.

### Concurrent and longitudinal associations with internalizing symptoms

Our findings regarding internalizing symptoms reveal a complex picture. Cross‐sectional analyses show that both lower levels of warmth and larger discrepancies in parental warmth, particularly when parents report higher warmth than their adolescents, are associated with increased adolescent internalizing symptoms. These results are supported by previous research highlighting that both the overall quality of the parent–adolescent relationship and the degree of discrepancy in perceptions contribute significantly to adolescent well‐being (De Los Reyes & Ohannessian, 2016; Nelemans et al., 2016). Discrepancies in perceptions of parenting can reflect a breakdown in parent–adolescent communication, with adolescents feeling misunderstood or emotionally disconnected, which in turn might exacerbate internalizing distress (De Goede et al., 2009). However, given the cross‐sectional nature of this relation, it is possible that internalizing symptoms themselves may contribute to adolescents' underestimation of parental warmth, further complicating our understanding of this dynamic (i.e., depression–distortion hypothesis; De Los Reyes & Kazdin, 2005; Richters, 1992).

We found evidence for a bidirectional longitudinal relation between warmth levels and internalizing symptoms. Specifically, lower levels of warmth predicted greater internalizing symptoms at a later time point, and conversely, adolescents experiencing higher internalizing symptoms reported lower warmth from their parents in subsequent assessments. This bidirectional pattern might suggest a dynamic interplay between emotional difficulties and parental warmth, where internalizing distress erodes parent–adolescent closeness, while diminished warmth further exacerbates adolescent psychological vulnerability, as posited in developmental psychopathology frameworks that emphasize recursive cycles of risk in parent–child relationships (Cicchetti & Toth, 2009). From a symptom‐driven perspective (Sameroff, 2009), these findings indicate that adolescents' negative emotional states can disrupt parent–child interactions over time. That is, emotional distress may contribute to parental withdrawal or exacerbate family conflict, thus resulting in a decrease in parental warmth. As previous research has consistently shown, mental health problems in adolescents can put a strain on parent–adolescent relationships and lead to a negative cycle of mutual disengagement (Hou et al., 2018; Nelemans et al., 2023).

Findings from the PAC study have provided robust evidence for these bidirectional effects, showing that adolescent adjustment problems predict decreases in positive parenting practices over time (Lansford et al., 2018) and that earlier parenting difficulties predict subsequent adolescent internalizing problems (Rothenberg et al., 2020). These bidirectional effects also appear in different cultural contexts (Rothenberg et al., 2022), which indicates that these developmental cascades may represent culture‐general processes in parent–adolescent relationships.

Longitudinal analyses did not reveal significant associations between discrepancies and internalizing symptoms in either direction. Although discrepancies may act as a short‐term stressor, signaling immediate emotional distress, they do not necessarily predict or are predicted by long‐term psychological maladjustment. This finding supports studies that have employed advanced statistical techniques, such as latent congruence models (Nelemans et al., 2023), which found that overall relationship quality is a better predictor of adolescent internalizing symptoms over time than the discrepancies themselves. However, it should be acknowledged that the lack of longitudinal effects may also be due to the complexity of the developmental trajectories involved, which means that discrepancies may not have a direct effect on long‐term adjustment but function differently in the context of other relational dynamics, such as family communication patterns, mutual expectations, and social and environmental factors (Cicchetti & Toth, 2009; Collins & Laursen, 2004).

These findings underscore the importance of considering both the general quality of the parent–adolescent relationship and discrepancies between parents' and adolescents' perceptions in relation to adolescent psychological outcomes over time. Additional longitudinal research should explore these dynamic relations, thereby enhancing our understanding of their influence on the developmental trajectories of well‐being and mental health in adolescents. Also, future research identifying the causes of discrepancies in parent–adolescent perceptions could improve and guide clinical efforts to reduce internalizing symptoms in adolescents. Interventions that address these gaps in parent–adolescent perceptions of family functioning might be crucial for understanding their role in the development or worsening of internalizing symptoms. Clinically, these findings underscore the importance of focusing on both the quality of the parent–adolescent relationship and the alignment of their perceptions. Clinicians should work to improve communication and warmth within families, enhancing parents' understanding of their adolescents' emotional needs. A holistic, relationship‐based approach that fosters open dialogue and addresses both internalizing symptoms and parenting practices may help promote adolescent well‐being.

### Cross‐cultural effects

Our study highlights cross‐cultural variations in both parental warmth levels and parent–adolescent discrepancies—an area that has been less explored than warmth levels alone. Previous research from the PAC study has shown that cultural norms shape parental warmth (Bornstein et al., 2021; Lansford et al., 2014, 2021); our findings extend this work by examining differences in how parents and adolescents perceive warmth across cultures.

For maternal warmth, lower levels were reported in China, Kenya, Thailand, and Jordan, while higher levels were found in Naples, Sweden, Colombia, and the United States (both Black and white U.S. groups). A similar trend emerged for paternal warmth, with lower levels in China, Rome, Kenya, Thailand, and Jordan, and higher levels in Sweden, Colombia, and all U.S. groups. Overall, these patterns align with previous research showing that collectivist societies tend to emphasize implicit expressions of care, whereas Western and some Latin American cultures tend to prioritize open affection (Bornstein et al., 2021). Colombia's high warmth levels, despite its collectivist orientation, indicate how Latin American familismo and emotional expressiveness can coexist with collective values. Sweden's high scores align with its low power distance and motivation toward achievement and success, while consistent patterns across diverse U.S. cultural groups may reflect shared exposure to individualistic values prioritizing explicit emotional expression. The difference between Rome and Naples is indicative of meaningful within‐country variation, with Naples' higher scores potentially reflecting regional differences in emotional expressiveness within Italian culture, with Southern Italy's regions tending to display greater emotional expressiveness.

Beyond absolute levels, we found cultural differences in parent–adolescent discrepancies that likely reflect distinct cultural models of family relationships and communication. Larger discrepancies in more collectivist cultures such as the Philippines may stem from hierarchical family structures where clear generational boundaries and respect for parental authority may create systematically different perspectives on the same interactions—parents may view their behavior as appropriately authoritative while adolescents may perceive it as more controlling, reflecting their different positions within the family hierarchy (Alampay & Rothenberg, 2021). Conversely, lower discrepancies in more individualist White U.S. families may reflect more egalitarian parent–child relationships and direct communication styles that promote shared understanding and perspective‐taking (Smetana, 2010).

Particularly noteworthy were the differences in the patterns of discrepancies in father–adolescent warmth in Thailand and Colombia. Father–adolescent dyads in Thailand showed lower discrepancies than those in other countries, which may be because, in traditional Thai families, fathers are primarily expected to provide financial support while mothers take the major responsibility of caregiving (Pummanee et al., 2018; Tapanya, 2011). This role division may create more emotionally reserved father–child relationships where both parties hold similar expectations about paternal warmth, leading to more similar ratings of perceived parental warmth. Conversely, Colombian father–adolescent dyads demonstrated significantly higher discrepancies, possibly reflecting the complex interplay between traditional machismo and modern paternal involvement in Latino cultures (Di Giunta et al., 2011). This pattern may be intensified by Colombia's rapid social transitions, where traditional and modern parenting approaches coexist within the same cultural context, creating ambiguity about appropriate father–child emotional expression and potentially leading fathers and adolescents to interpret the same behaviors through different cultural lenses (Carrillo et al., 2016).

Together, these results suggest that discrepancies between parents and adolescents in terms of warmth perceptions may represent culturally shaped patterns rather than a sign of dysfunction and thus support the need to consider cultural relevance in family relationship assessment and adolescent development studies (Bornstein, 2012; Trommsdorff & Kornadt, 2003). Overall, our research further suggests that while studying warmth levels is important, it is also necessary to examine variations in perceptions of warmth across cultures because these might be associated with particular parenting practices that might have certain implications for adolescent development.

### Limitations and future directions

This study has several limitations. First, our assessment of warmth was based on questionnaire measures, which may not capture the full extent of parent–adolescent interactions. Although IPARTheory emphasizes subjective perceptions of parental warmth, it is important to acknowledge that self‐report measures can be influenced by biases related to mood, memory, and social desirability. Such biases may produce discrepancies that do not accurately reflect actual behaviors. Also, a potential limitation arises from the shared variance between adolescents' reports of parental warmth and their internalizing symptoms. Since both variables were self‐reported by the same individuals, this shared variance could inflate the observed correlations, potentially amplifying the strength of the association between perceived warmth and internalizing symptoms. Future research could benefit from the use of observational or daily diary methods to provide a more detailed view of how warmth is displayed in parent–adolescent interactions on a daily basis.

Furthermore, individuals' affect can influence parent and adolescent reports of parenting behaviors (Janssen et al., 2023), thus supporting the importance of taking emotional states into account when interpreting discrepancies in adolescent–parent reports, particularly in clinical and research settings. Second, although our study examined bidirectional relations over time, the relatively long measurement intervals may have hidden short‐term fluctuations in warmth and discrepancies. More frequent assessments, including ecological momentary assessment (EMA), could help capture the dynamic interplay between adolescent internalizing symptoms and parent–adolescent relationship quality. Such methodologies could provide insights into how these relationships manifest in real time and how transient emotional states shape adolescents' perceptions of parental warmth. Additionally, future research should explore potential moderators of these associations, such as family communication patterns or cultural values, which may shape how discrepancies emerge and the implications they have for adolescent adjustment.

Expanding on the cross‐cultural dimension, our study highlights meaningful variations in parent–adolescent perceptions of parental warmth across cultures. However, due to the limited size of our culture‐specific samples, further research is needed to explore how these discrepancies manifest, how they relate to adolescent adjustment, and how they manifest in multicultural and immigrant families, where values shaping parenting behaviors in the host country may differ from those in the country of origin. Acculturation processes, particularly the extent to which parents and adolescents adopt the cultural norms of the host society, may play a crucial role in shaping parental warmth and its perception. Prior research has shown that discrepancies in acculturation between parents and adolescents—where adolescents integrate host culture values more rapidly than their parents—can create tensions that affect perceived parental warmth and relationship quality (Bornstein, 2017; Bornstein & Lansford, 2019). Investigating how cultural adaptation influences parent–adolescent warmth and whether discrepancies in perceptions of parental warmth are different for first‐ and second‐generation immigrant families could help to understand the role of cultural context in shaping parent–child dynamics.

Finally, an important direction for future research is to examine the mechanisms through which discrepancies in warmth perception can influence relationship quality and adolescent adjustment. Understanding whether discrepancies arise from poor communication between parents and adolescents, different expectations that parents and adolescents have concerning the parent–adolescent relationship, or for autonomy, for instance, or from more severe dysfunctions of the relational system is crucial for developing interventions to improve parent–adolescent relationships. These gaps can be addressed through multimethod approaches and cross‐cultural comparisons to provide a more holistic view of how parent–adolescent warmth contributes to adolescent well‐being across diverse family contexts.

Despite its limitations, the present study offers several key strengths, which extend existing research on parent–adolescent discrepancies in perceptions of relationship quality and in adolescent adjustment. By integrating the IPARTheory, the study provides a robust theoretical framework emphasizing subjective perceptions of parental warmth, making it particularly suitable for cross‐cultural comparisons. In addition, the use of latent congruence models allows for a nuanced examination of both mean levels and discrepancies in parent and adolescent perceptions, thus offering a unique approach to studying the bidirectional relations between warmth discrepancies and adolescent internalizing symptoms. Finally, the study's cross‐cultural scope, incorporating diverse cultural contexts, sheds light on the contextual factors that might influence parent–adolescent discrepancies in reports of parental warmth, underscoring the need for further research to explore how these cultural differences shape the associations of parent–adolescent relationships with adolescent adjustment.

## CONCLUSION

The present study contributes to this literature by examining relations between these discrepancies and adolescent adjustment, considering both adolescent–maternal and paternal relationships. Mothers rate themselves as warmer than their adolescents do, whereas father–adolescent relationships show more complexity in terms of warmth perceptions. Higher parental warmth, in both mother‐ and father–adolescent dyads, was associated with lower internalizing symptoms, and higher internalizing symptoms were associated with lower warmth in subsequent assessments, suggesting a bidirectional relation. Although cross‐sectional associations between warmth discrepancies and adolescent well‐being were significant, these discrepancies appeared not to drive long‐term changes in adolescent internalizing symptoms. Furthermore, discrepancies in perceived warmth may reflect culturally specific parenting practices, which supports the need for a contextualized approach when examining family dynamics.

From a clinical perspective, fostering alignment between parents and adolescents in their perceptions of parental behavior can enhance the quality of parent–adolescent interactions, leading to a deeper understanding and reduced misunderstandings within the dyad during this critical developmental stage. Additionally, encouraging open, supportive, and empathic communication between parents and adolescents can serve to mitigate detrimental effects that misunderstandings may evoke. By creating a safe space for dialogue, both parties can express their thoughts and feelings more freely, ultimately strengthening the parent–adolescent relationship.

However, achieving these goals requires approaches that are culturally responsive and sensitive to the values and norms that shape family relationships and the effectiveness of interventions (Lansford, 2022; Mejia et al., 2017). As Lansford (2022) underscores, interventions transported from one cultural context to another benefit from careful adaptation to local preferences, customs, beliefs, and prohibitions, along with ethical reflection on how efforts to change parenting practices may affect particular cultural groups. In collectivist cultures, for instance, fostering open communication must be balanced with respect for hierarchical family structures. Filipino families illustrate this complexity: parenting tends to emphasize strictness, respect for authority, and obedience, reflecting a more authoritarian orientation (Alampay, 2014). Although nearly half of Filipino youth report that they do not view physical or psychological punishment as problematic—suggesting a cultural normativeness of these practices—research shows that children nevertheless experience hurt, anger, sadness, and fear in response to harsh discipline (Council for the Welfare of Children and UNICEF Philippines, 2016). This illustrates the critical challenge facing culturally responsive interventions: how to respect cultural norms while addressing practices that may be harmful to child development, as the consequences of harsh physical punishment remain demonstrably negative even among Filipino samples (Alampay et al., 2017). Such contexts require interventions that work within existing cultural frameworks while gradually promoting alternative approaches, as exemplified by initiatives like PLH‐Philippines (Alampay et al., 2018), which addresses these challenges through culturally adapted evidence‐based parenting programs.

Building on this idea, family‐based interventions can be tailored to different cultural orientations. In collectivist contexts, they might focus on creating structured opportunities for respectful dialogue—for example, culturally guided family meetings where parents preserve their respected role while becoming more emotionally accessible, and adolescents can share their perspectives in ways that uphold family hierarchy. In contrast, in individualist cultures, interventions can more directly emphasize egalitarian communication, perspective‐taking, and collaborative problem‐solving. Parent education programs that foster active listening and validate adolescents' needs for autonomy may also be readily integrated into existing school‐based or clinical services.

Addressing socioeconomic barriers is equally crucial across all cultural contexts (Mejia et al., 2015). In lower resource settings, community health workers and peer educators can deliver culturally adapted programs using accessible group formats that are tailored to the needs of the community. Importantly, such barriers exist not only between countries but also within them, as low‐income families in high‐resource nations may face challenges more similar to those in low‐resource settings. Conditional cash transfer programs can further support families by directly reducing economic strain while incentivizing health, education, and parenting engagement. In higher‐resource contexts, family therapy and school‐based prevention programs may be more feasible. Universal elements should include psychoeducation about adolescent development, training in perspective‐taking, and conflict management skills, but delivery methods must be tailored to match each culture's communication norms, family structures, and available resources.

Research on the association between parent–adolescent discrepancies in perceptions of parental warmth and adolescent internalizing symptoms has produced mixed findings, suggesting the need to explore this complex dynamic further. Future research should aim to understand how these processes evolve in different cultural contexts and at various developmental stages, thereby expanding our knowledge about complex relations between dyadic subjective perceptions and any discrepancies in family functioning that may arise between parents and their adolescents, as well as the impact on adolescents' mental health.

## AUTHOR CONTRIBUTIONS

**Maria Concetta Miranda:** Investigation; writing – review and editing. **Concetta Esposito:** Conceptualization; writing – original draft; formal analysis. **W. Andrew Rothenberg:** Writing – review and editing. **Paul Oburu:** Investigation; writing – review and editing. **Emma Sorbring:** Investigation; writing – review and editing. **Daranee Junla:** Investigation; writing – review and editing. **Sevtap Gurdal:** Investigation; writing – review and editing. **Concetta Pastorelli:** Investigation; writing – review and editing. **Jennifer E. Lansford:** Funding acquisition; writing – review and editing. **Saengduean Yotanyamaneewong:** Investigation; writing – review and editing. **Liliana Maria Uribe Tirado:** Investigation; writing – review and editing. **Laura Di Giunta:** Investigation; writing – review and editing. **Ann T. Skinner:** Data curation; investigation; writing – review and editing. **Kirby Deater‐Deckard:** Writing – review and editing. **Suha M. Al‐Hassan:** Investigation; writing – review and editing. **Liane P. Alampay:** Investigation; writing – review and editing. **Laurence Steinberg:** Writing – review and editing. **Marc H. Bornstein:** Writing – review and editing. **Lei Chang:** Investigation; writing – review and editing. **Kenneth A. Dodge:** Writing – review and editing. **Dario Bacchini:** Investigation; supervision; writing – review and editing.

## FUNDING INFORMATION

This research has been funded by the Eunice Kennedy Shriver National Institute of Child Health and Human Development (Grant no. R01‐HD054805) and the Fogarty International Center (Grant no. R03‐TW008141). This research was also supported by the H2020 European Research Council (ERC) (Grant no. 695300‐HKADeC‐ERC‐2015‐AdG).

## CONFLICT OF INTEREST STATEMENT

The authors have no known conflicts to disclose.

## ETHICS STATEMENT

The study was approved by the local institutional review boards of collaborating universities in each country.

## INFORMED CONSENT

Informed consent was obtained from adult participants, and assent was obtained from youth participants.

## Supporting information


Appendix S1.

## Data Availability

The data that support the findings of this study are available from the corresponding author upon reasonable request.
