# Supplementary material for: Parent–adolescent discrepancies in perceptions of parental warmth: Cross‐cultural differences and longitudinal associations with internalizing symptoms
Source: J Res Adolesc. 2025 Oct 31;35(4):e70093. doi: 10.1111/jora.70093 (PMC12576307; doi:10.1111/jora.70093)
Supplement: Supplementary file 1 — Appendix S1. [file JORA-35-0-s001.docx]

**Supplemental Material**

**Supplementary figures**

**Figure S1**

Scatterplots Displaying Significant Associations Involving Discrepancy Scores. (a) Association between Discrepancy and Mean Level of Warmth in Mother-Adolescent Dyads; (b) Association between Mother-Adolescent Discrepancy and Internalizing Symptoms; (c) Direct Effect of Wave 1 Father-Adolescent Discrepancy on Wave 2 Mean Level of Warmth; (d) Association between Discrepancy and Mean Level of Warmth in Father-Adolescent Dyads; (e) Association between Father-Adolescent Discrepancy and Internalizing Symptoms.


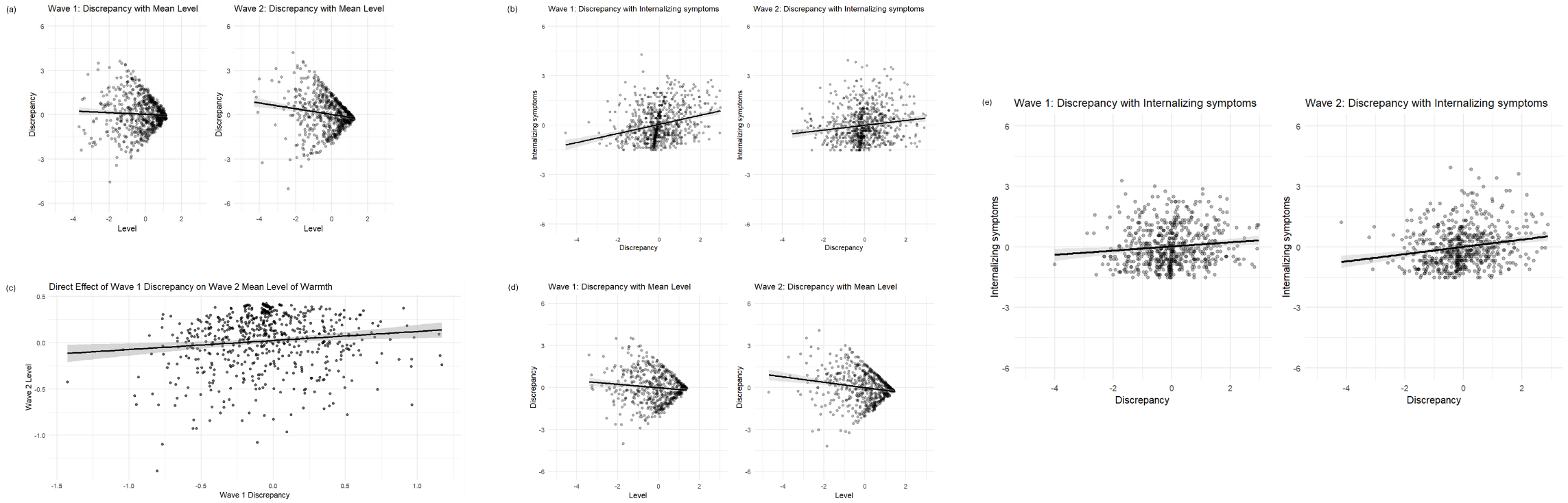


**Supplementary tables**

**Table S1**
*Means and Standard Deviations for Maternal Warmth, Mother–Child Discrepancy, and Internalizing Symptoms by Cultural Group across Waves*

| Cultural Group |  | Wave 1 | | |  | Wave 2 | | |  | Wave 3 |
| --- | --- | --- | --- | --- | --- | --- | --- | --- | --- | --- |
|  |  | Maternal warmth | Mother-child discrepancy | Internalizing symptoms |  | Maternal warmth | Mother-child discrepancy | Internalizing symptoms |  | Internalizing symptoms |
| China |  | 0.06 (0.18) | 0.15 (0.24) | 11.28 (6.53) |  | 0.05 (0.22) | 0.06 (0.29) | 8.89 (7.47) |  | 7.61 (7.12) |
| Italy-Naples |  | 0.19 (0.18) | 0.17 (0.23) | 16.07 (7.82) |  | 0.20 (0.19) | 0.12 (0.27) | 12.36 (6.88) |  | 13.42 (8.41) |
| Italy-Rome |  | 0.18 (0.17) | 0.20 (0.23) | 14.37 (8.31) |  | 0.13 (0.23) | 0.18 (0.25) | 11.69 (7.57) |  | 13.37 (7.83) |
| Kenya |  | -0.06 (0.26) | 0.19 (0.25) | 9.18 (6.40) |  | 0.12 (0.22) | 0.00 (0.27) | 7.71 (3.36) |  | 13.70 (8.86) |
| Philippines |  | 0.20 (0.19) | 0.22 (0.28) | 18.93 (8.12) |  | 0.14 (0.23) | 0.20 (0.28) | 18.84 (8.01) |  | 18.57 (9.10) |
| Thailand |  | -0.01 (0.27) | 0.26 (0.29) | 13.89 (8.11) |  | -0.05 (0.29) | 0.16 (0.34) | 14.95 (8.38) |  | 14.77 (8.33) |
| Sweden |  | 0.22 (0.19) | 0.18 (0.22) | 13.17 (8.29) |  | 0.22 (0.17) | 0.11 (0.21) | 9.12 (6.57) |  | 8.96 (6.31) |
| U.S. Black |  | 0.24 (0.18) | 0.16 (0.27) | 15.21 (9.05) |  | 0.22 (0.19) | 0.12 (0.29) | 11.03 (7.64) |  | 12.12 (8.97) |
| U.S. White |  | 0.29 (0.12) | 0.13 (0.18) | 14.56 (6.89) |  | 0.27 (0.15) | 0.08 (0.20) | 12.21 (7.61) |  | 13.29 (9.52) |
| U.S. Latino |  | 0.21 (0.22) | 0.12 (0.26) | 15.68 (8.33) |  | 0.21 (0.22) | 0.11 (0.24) | 11.01 (8.77) |  | 9.07 (6.84) |
| Colombia |  | 0.23 (0.17) | 0.14 (0.24) | 19.26 (9.67) |  | 0.17 (0.28) | 0.16 (0.24) | 10.76 (6.71) |  | 14.22 (8.65) |
| Jordan |  | 0.12 (0.22) | 0.11 (0.28) | 13.90 (7.73) |  | 0.03 (0.27) | 0.09 (0.26) | 11.71 (7.23) |  | 13.84 (7.80) |
| **Whole sample** |  | **0.15 (0.22)** | **0.17 (0.25)** | **14.65 (8.39)** |  | **0.14 (0.24)** | **0.12 (0.27)** | **11.74 (7.79)** |  | **12.90 (8.65)** |

*Note*. Standard deviations are in parentheses. Factor scores for maternal warmth and mother–child discrepancy are standardized estimates (Mean ≈ 0, SD ≈ 1). Internalizing symptoms are presented as raw scaled means.

**Table S2***Means and Standard Deviations for Paternal Warmth, Father–Child Discrepancy, and Internalizing Symptoms by Cultural Group across Waves*

| Cultural Group |  | Wave 1 | | |  | Wave 2 | | |  | Wave 3 |
| --- | --- | --- | --- | --- | --- | --- | --- | --- | --- | --- |
|  |  | Paternal warmth | Father-child discrepancy | Internalizing symptoms |  | Paternal warmth | Father-child discrepancy | Internalizing symptoms |  | Internalizing symptoms |
| China |  | -0.11 (0.30) | -0.05 (0.42) | 8.89 (7.47) |  | -0.10 (0.28) | 0.00 (0.35) | 7.61 (7.12) |  | 10.55 (7.26) |
| Italy-Naples |  | 0.05 (0.28) | 0.00 (0.36) | 12.42 (6.93) |  | 0.02 (0.30) | 0.04 (0.36) | 13.29 (8.43) |  | 16.06 (9.85) |
| Italy-Rome |  | -0.03 (0.26) | -0.13 (0.36) | 11.85 (7.56) |  | -0.10 (0.31) | 0.08 (0.36) | 13.29 (7.84) |  | 15.03 (8.96) |
| Kenya |  | -0.29 (0.26) | 0.07 (0.37) | 7.71 (3.36) |  | 0.04 (0.25) | -0.03 (0.34) | 13.80 (8.87) |  | 13.92 (6.81) |
| Philippines |  | 0.00 (0.25) | 0.09 (0.34) | 19.49 (7.76) |  | 0.01 (0.24) | 0.07 (0.30) | 18.84 (9.41) |  | 18.37 (8.44) |
| Thailand |  | -0.14 (0.29) | -0.11 (0.39) | 14.87 (7.86) |  | -0.21 (0.33) | -0.01 (0.44) | 14.77 (8.33) |  | 15.82 (9.20) |
| Sweden |  | 0.17 (0.18) | -0.12 (0.26) | 8.87 (6.17) |  | 0.12 (0.21) | -0.05 (0.29) | 8.70 (6.24) |  | 10.73 (8.31) |
| U.S. Black |  | 0.16 (0.20) | -0.11 (0.27) | 10.59 (7.66) |  | 0.09 (0.24) | -0.04 (0.38) | 12.22 (8.99) |  | 12.39 (10.04) |
| U.S. White |  | 0.19 (0.19) | -0.14 (0.25) | 12.39 (7.67) |  | 0.12 (0.21) | -0.08 (0.25) | 13.24 (9.62) |  | 16.21 (11.11) |
| U.S. Latino |  | 0.13 (0.24) | -0.05 (0.27) | 10.89 (8.70) |  | 0.09 (0.24) | -0.02 (0.35) | 8.68 (6.36) |  | 11.96 (8.21) |
| Colombia |  | 0.15 (0.25) | 0.01 (0.31) | 10.74 (6.74) |  | 0.08 (0.26) | 0.14 (0.35) | 14.30 (8.68) |  | 17.86 (11.77) |
| Jordan |  | -0.04 (0.30) | 0.01 (0.35) | 11.76 (7.25) |  | -0.10 (0.35) | 0.02 (0.36) | 13.83 (7.83) |  | 12.29 (8.73) |
| **Whole sample** |  | **0.01 (0.29)** | **-0.04 (0.34)** | **11.71 (7.73)** |  | **0.00 (0.29)** | **0.01 (0.35)** | **12.86 (8.69)** |  | **14.51 (9.51)** |

*Note*. Standard deviations are in parentheses. Factor scores for paternal warmth and father–child discrepancy are standardized estimates (Mean ≈ 0, SD ≈ 1). Internalizing symptoms are presented as raw scaled means.
